# Early clinical and radiological evaluation in patients with total ankle replacement performed by lateral approach and peroneal osteotomy

**DOI:** 10.1186/s12891-019-2503-6

**Published:** 2019-03-27

**Authors:** Alberto Bianchi, Nicolò Martinelli, Mohammad Hosseinzadeh, Jacopo Flore, Carlo Minoli, Francesco Malerba, Fabio Galbusera

**Affiliations:** 1 IRCCS Istituto Ortopedico Galeazzi, Via R. Galeazzi 4, 20100 Milan, Italy; 2 Department of Orthopedics, University of Milan, Milan, Italy; 3 Department of Ankle and Foot Surgery, IRCCS Istituto Ortopedico Galeazzi, Via R. Galeazzi 4, 20100 Milan, Italy

**Keywords:** Ankle arthroplasty, Ankle osteoarthritis, Pain, Total ankle replacement

## Abstract

**Background:**

The Zimmer Trabecular Metal Total Ankle Replacement (Zimmer TM TAR) is a recent ankle arthroplasty approved for use in the United States and Europe. Many of the studies reporting the results of this implant are provided by surgeons involved at least in the initial design of the implant under study.

The aim of this study is to describe the early clinical and radiological outcomes in patients who underwent this procedure performed by non-designer surgeons.

**Methods:**

A total of thirty consecutive patients underwent total ankle replacement with a Zimmer TM TAR surgery between July 2013 to January 2016.All clinical assessments were collected pre- and post-operatively with minimum follow-up of 12 months for each patient using the American Orthopedic Foot and Ankle (AOFAS) score, the Foot Function Index (FFI) and a visual analogue scale (VAS) for pain. Radiographic outcomes included ankle orientation assessed with angle “α”,“β” and “γ” according to Wood. Furthermore, the anteroposterior offset ratio was measured in weight-bearing lateral ankle radiographs at the last follow-up.

**Results:**

The mean preoperative FFI-pain (FFI-P) value was 53.67, the FFI-disability (FFI-D) was 64.19. At the last follow-up visit, the FFI-P and FFI-D was 16.95 and 20.76 respectively (*p*<0.01 for the both scales). Preoperatively, the mean VAS for pain and AOFAS score was 7.81 and 40.95 respectively, and at the last follow-up 2.29 and 86.38 (*p*<0.01 for the both scales). The mean angle calculated using Wood and Deakin’s method were “α”= 89.02°, “β”= 85.11 and “γ”= 27.54 post-operatively. At the last follow-up the same values were respectively 89.43, 85.18 and 29.94. At the last follow-up, the mean offset ratio was 0.06 (range 0.003/-0.17).

**Conclusions:**

These early results show high levels of patient satisfaction, and we are encouraged to continue with lateral approach total ankle arthroplasty.

## Background

Numerous studies reported the efficacy of new third-generation of total ankle arthroplasties for the treatment of advanced ankle osteoarthritis with clinical results comparable to ankle arthrodesis [1, 2]. The improvement in knowledge of ankle joint biomechanics, instrumentation, surgical approach may led to even higher clinical results in comparison to arthrodesis. The Zimmer Trabecular Metal Total Ankle Replacement (Zimmer TM TAR) is a recent ankle arthroplasty approved for use in the United States and Europe. The implant requires a lateral approach and a peroneal osteotomy. Potential advantages with a trans-fibular approach included the direct visualization of the center of rotation of the ankle and a theoretical reduced risk of wound complications due to exposure of the ankle joint through a surgical plane between angiosomes. Furthermore, this approach allows the surgeon to address coronal rotational and sagittal plane and provides a more physiological orientation of the tibia and talar component.

Many of the studies reporting the results of this implant are provided by surgeons involved at least in the initial design of the implant under study [3]. Thus, familiarity with the system can be a potential bias affecting outcomes and orthopedic surgeons may find difficulties in reproducing the results.

The aim of this study is to describe the early clinical and radiological outcomes in patients who underwent this procedure performed by non-designer surgeons.

### Methods

This retrospective study was approved by our ethical committee (San Raffaele Hospital, 76/int/2017) and a written informed consent was obtained from all patients.

A group of thirty consecutive patients who underwent total ankle replacement with a Zimmer TM TAR surgery between July 2013 to January 2016 was unrolled in this study. Indications for TAR were end-staged osteoarthritis of the ankle (primary, secondary, or post-traumatic) with good bone stock, neutral alignment or mild-to-moderate malalignment, good stability, and preserved motion of the ankle. Preoperative hindfoot malalignment was not considered an absolute contraindication, but additional surgical steps (supramalleolar and/or calcaneal osteotomies, ligament reconstruction, subtalar arthrodesis) was performed to correct the deformity.

There were 11 women and 19 men. The mean age was 58.2 years (range 22-77). The mean follow-up was 29.7 months (range 18-49).

Indications for surgery were mainly represented by post-traumatic osteoarthritis, secondary ankle osteoarthritis to giant cell tumor of the tendon sheath (GCT-TS), one rheumatoid arthritis and one primary osteoarthritis. Fifteen patients had post-traumatic osteoarthritis secondary to ankle fracture while eleven patients had post-traumatic osteoarthritis secondary to ligament injuries. The patient with ankle osteoarthritis secondary to GCT-TS category refused an ankle fusion, and even though he had 22 years at surgery, we performed tumour removal and TAR. Routinely, we are against TAR in young adult patients, but the strong will of the patient forced us to change indication. The patient was also fully informed about the theoretical survivorship of the implant and the possible future treatments in case of failure or GCT-TS recurrence.

Patients with neuropathy, diabetes, excessive bone loss (>50%) due to avascular necrosis or trauma, recent history of septic arthritis or obese (BMI>30kg/m [2]) were considered not suited for this type of surgery and therefore underwent different treatments.

Exclusion criteria included incomplete records, a lack of adequate follow-up duration and the use of different implants.

All clinical assessments were collected pre- and post-operatively with minimum follow-up of 12 months from each patient using the American Orthopedic Foot and Ankle Society (AOFAS) score, the Foot Function Index (FFI) and the visual analogue scale (VAS) for pain [4, 5]. Patients were also asked to assess their satisfaction with the surgical outcome based on a three-grade scale: very satisfied, satisfied or not satisfied with the result. The range of movement (ROM) was evaluated using a goniometer by one of the investigators (JF).

**Fig. 1:**
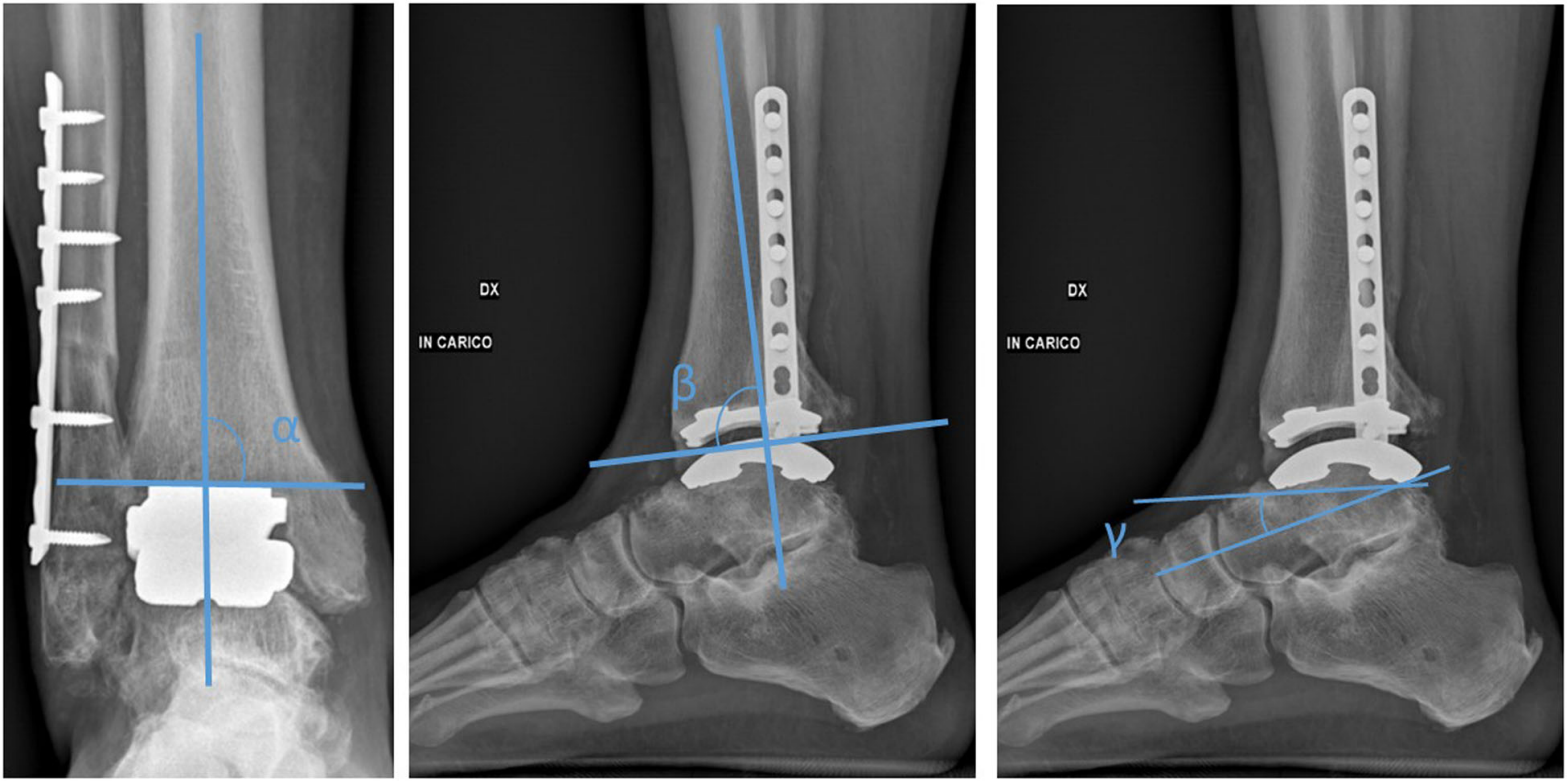
Radiographic angles (α, β, γ) for assessment of components position and migration

Standard (anterior-posterior and lateral) weightbearing radiographs of the ankle were collected pre-operatively and at the last follow-up for all patients and independently evaluated by two orthopedic surgeons (AB, JF). These radiographs were assessed for the presence of aseptic loosening (i.e. radiolucency > 2 mm in width, bone cysts at the margin of a component or migration of a component [6]). The components alignment was checked with the method by Wood and Deakin, either post-operatively and at the last follow-up using three angular measurements (α, β and γ) (Fig. 1) [7].

A change in positions bigger than 3° relative to the long axis of the talus, assessed on the lateral radiograph (“γ”) was considered sign of loosening. Furthermore, the anteroposterior offset ratio was measured in weight-bearing lateral ankle radiographs at the last follow-up [8]. Positive values were defined when the center of the circle was anterior to the tibial axis and negative values when posterior to the tibial axis.

### Surgical technique

The surgical technique was carried out following to the manufacturer’s guidelines with the original Zimmer TM TAR instrumentation. An oblique osteotomy (superolateral to inferomedial) starting approximately 2.5 cm proximally to the ankle joint line is performed. The anterior talofibular ligament (ATFL) is sectioned sparing enough tissue to perform reconstruction at the end of the implantation. The posterior capsule and ligaments are then released. Osteophytes in the medial gutter and any anterior osteophytes can be managed through a small incision medial to the anterior tibial tendon. The leg is then placed in the alignment frame and the alignment is checked on anteroposterior and lateral fluoroscopy. The bone resection is completed using the cutting guide and a rotational burr. The prosthesis is implanted according to previous intraoperative measurements with regard to prosthesis size in both frontal and sagittal planes. Fibular osteotomy is then fixed with plate and screws. After fibular fixation, the ATFL was repaired. After surgery a short leg cast is made, and weight-bearing is prohibited for 1 month. At the end of this period the cast is removed and partial weight-bearing is allowed for 2 weeks. At 6 weeks from surgery weight-bearing is allowed without restrictions and rehabilitation with a physiotherapist is encouraged.

### Statistical analysis

A Student’s t-test or a Wilcoxon’s test were used to assess differences in the pre-operative and follow-up clinical scores (AOFAS score and VAS for pain), after a normality test (i.e. Kolmogorov-Smirnoff Z test). The statistical significance level was set at p < 0.05. Analyses were performed using SPSS 20.0 for Windows (SPSS Inc., Chicago, Illinois).

## Results

Thirty consecutive patients underwent total ankle replacement with a Zimmer TM TAR surgery between July 2013 to January 2016. At index procedure, 11 (37%) of cases included at least 1 concurrent procedure, and 19 cases did not include any concurrent procedures (63%) (Table 1).

**Table 1:** Concomitant procedures at primary total ankle arthroplasty

Procedure	Frequency
Tendo-Achilles lengthening	6
Hardware removal (from previous surgery)	3
Calcaneal osteotomy	1
Subtalar fusion	1
Other^a^	3

^a^Other procedures included open reduction and internal fixation (ORIF) medial malleolus for intraoperative fracture, excision of periarticular calcification, syndesmosis fixation

The mean preoperative FFI-pain (FFI-P) value was 53.67, the FFI-disability (FFI-D) was 64.19. At the last follow-up visit, the FFI-P and FFI-D was 16.95 and 20.76 respectively (*p*<0.01 for the both scales). Preoperatively, the mean VAS for pain and AOFAS score was 7.81 and 40.95 respectively, and at the last follow-up 2.29 and 86.38 *p*<0.01 for the both scales). The average postoperative tibiotalar ROM was 18.5 degrees in plantarflexion and 8 degrees in dorsiflexion.

The mean angle calculated using Wood and Deakin’s method were “α” 89.02°, “β” 85.11 and “γ” 27.54 post-operatively. At the last follow-up the same values were respectively 89.43, 85.18 and 29.94 (*p*> 0.05 for the angles). At the last follow-up, the mean offset ratio was 0.06 (range 0.003/-0.17). Ten percent was found to be slightly anterior to the tibial axis and 3.3 % (1 patient) posterior in the examined radiographs.

**Fig. 2:**
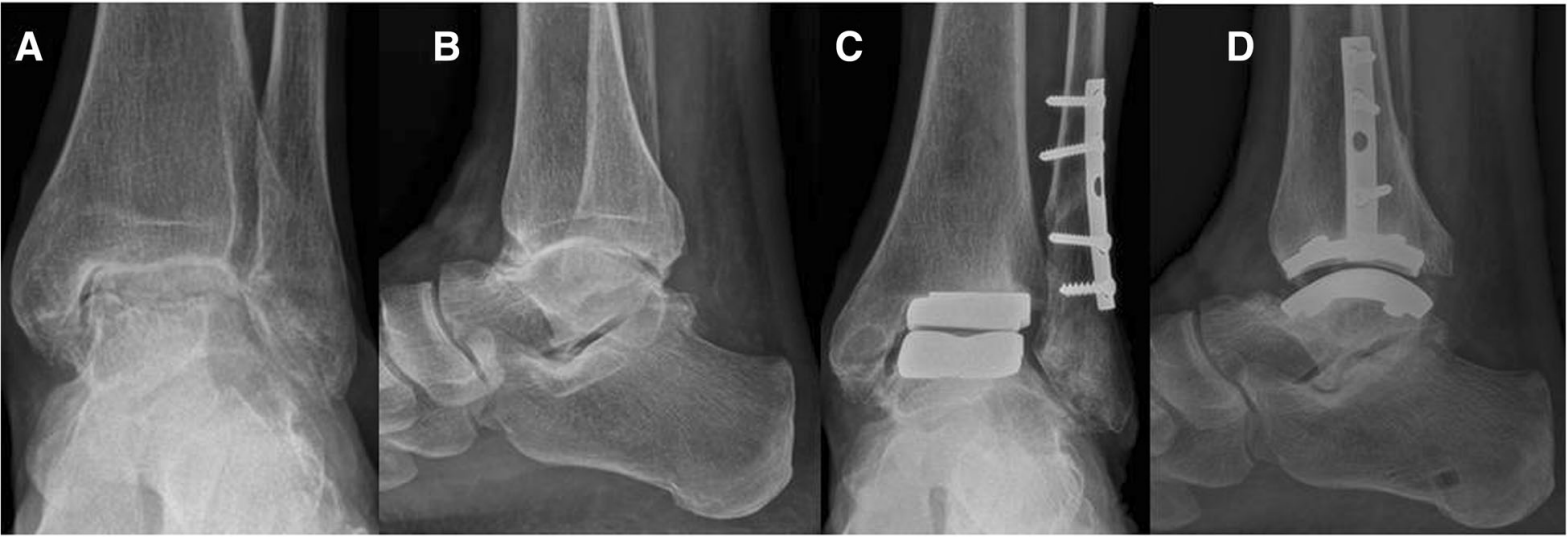
Pre-operative (**a**, **b**) and two-year follow-up weight bearing radiograph after Zimmer TM TAR showing a medial malleolar cyst (**c**, **d**)

When assessing radiographs for subsidence/loosening, most implants prosthesis showed no radiographic evidence of cyst and/or lucency on either AP and/or lateral views except for three patients with radiolucent lines in the anterior and posterior part of the tibia-prosthesis interface, one patient with radiolucent lines in the anterior part of the tibia-prosthesis interface and one patient with a medial malleolar cyst (Fig. 2).

Most patients (91%) were satisfied or very satisfied after surgery and would have repeat the surgery if under the same conditions. The rate of major complications requiring surgery was 23.3 % (7 patients).

**Fig. 3:**
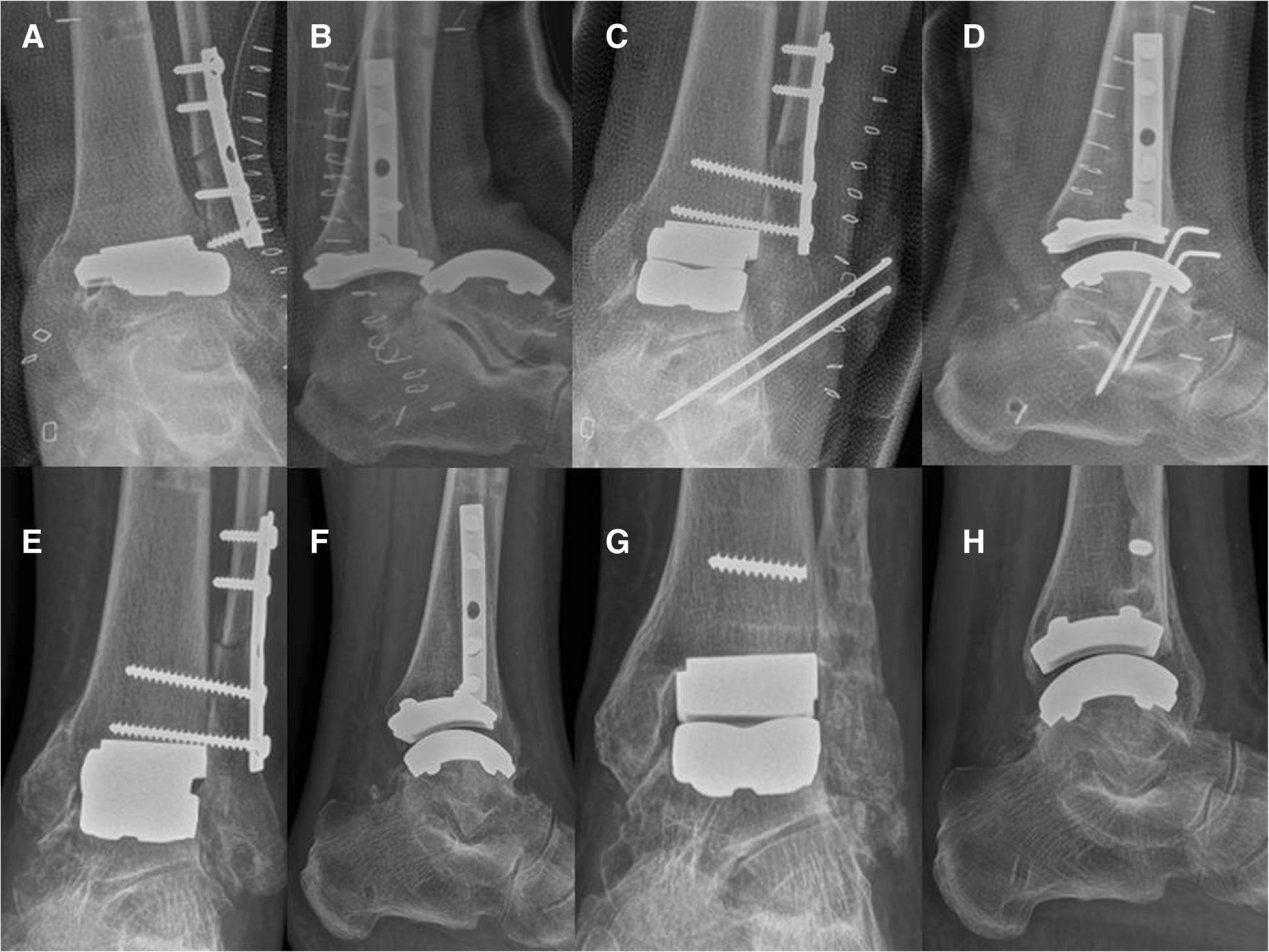
Dislocation of the Zimmer TM TAR (**a**, **b**) with a undiagnosed intra-operative medial malleolar fracture, reduction and temporal fixation (**c**, **d**), six months follow-up (**e**, **f**), one year follow-up (**g**, **h**)

One patient had a perioperative dislocation of the ankle (Fig. 3). Dislocation occurred the day after implantation, during plaster cast positioning. At the post-operative radiography after cast positioning, the ankle was dislocated. Treatment consisted in open reduction, syndemosis fixation and temporary fixation of the reduction with extrarticular kirschner wires. After reduction of the dislocation, we decided not to fix the medial malleolar since the construct was quite stable intra-operatively and because a non-weightbearing cast was applied for six weeks. The reason for dislocation was missed diagnosis of the medial malleolar fracture during definitive components positioning: we did not recognize the malleolar fracture at the last intra-operative radiographic control. Eighteen months after surgery the patient underwent removal of the fibular plate and screws for skin irritation. Two months after hardware removal the patient complained mild pain during walking and was satisfied with the outcome of the procedure.

Six patients underwent revision surgery. One patient developed a nonunion of the fibula that was therefore treated with autologous bone graft, plate fixation and medializing calcaneal osteotomy for residual hindfoot valgus. The patient did not present any factors for nonunion. A diagnosis of nonunion was made 7 months after surgery based on clinical and radiological features. Union was reached at three months after surgery. The second and third patient was still reporting pain on the lateral side of the ankle joint one year after TAR with a residual varus deformity of the hindfoot. We performed a calcaneal Z osteotomy with Malerba’s technique in the second patient and a Malerba’s osteotomy associated with a dorsiflexory wedge osteotomy at the base of the first metatarsal in the third patient. After three months the patients had a significant clinical improvement and were very satisfied with the surgery. The fourth and fifth patient underwent hardware removal (fibular plate) 12 and 14 months respectively, after the index surgery for skin irritation. The last patient underwent a medializing calcaneal osteotomy for symptomatic residual hindfoot valgus 13 months after TAR. The most common cause of hindfoot deformity correction after TAR was the pre-operative and intra-operative misjudgment of hindfoot alignment.

One patient developed an asymptomatic non-union, which did not require any further treatment but clinical and radiological follow-up.

One patient that had residual pain after surgery is currently pregnant and therefore, we are waiting, with her consent, before making further radiographical analysis and revisional surgery. She has no functional deficit and the clinic is not susceptible of failure of the implant, loosening or infection and therefore the wait and see strategy seems, in our opinion, reasonable.

## Discussion

Total ankle replacement is a common procedure with predictable results and is becoming increasingly more frequent for the treatment of ankle osteoarthritis. Registry data from different countries have shown an increase in the use of TAR for ankle osteoarthritis following traumatic event, rheumatoid arthritis and other conditions that cause a progressive loss of integrity of the tibio-tarsal joint [9].

In 2012 the Food and Drug Administration approved a new type of prothesis: the Zimmer Trabecular Metal Total Ankle (Zimmer Inc.,Warsaw, IN). This implant was designed to allow the implant trough lateral approach. The main objective was to introduce a prosthesis that could decrease surgical complication linked to delayed wound healing and to aid the surgeon who was more familiar with the lateral approach used for ankle arthrodesis [10]. Furthermore, this model is equipped with trabecular metal material, which was developed to simulate the normal architecture of the cancellous bone. Bobyn et al. have shown that the porous structure of this material permits bone ingrowth in most of the available surface, thus increasing the stability [11]. Even though the prosthesis in the U.S. is implanted with cement fixation, it is commonly used off-label without cement and fixation relies on bony ingrowth at the bone-implant interface.

The risk of wound healing issues is present in 2-40% of cases of TAR performed throw anterior approach [12, 13]. Early TAR procedures had problems with wound healing but improvements in techniques, materials and postoperative care have reduced the incidence of infections. The percentage of incidence throw lateral approach should be theoretically lower due to the incision lying between two angiosomes and therefore causing a minor vascular damage of the skin.

The other main advantage of a lateral approach is the possibility to have a direct visualization of the rotational center of the joint, and therefore allowing a more accurate bone resection and reconstruction of the ankle joint anatomy and angles.

Some authors report the possibility of a major stability of the components due to the bone perpendicular orientation of the tibial and talar trabeculae that may improve the force transfer from implant and decrease the shear forces at the bone-implant interface [3]. Those data however, don’t have a statistical proof at the moment.

The complications of a lateral approach are associated to the fibular osteotomy with subsequent risk of delayed union, nonunion, change in fibular length, vascular and neural damage and painful hardware. Tennant et al reported the risk of lesion to the perforator peroneal artery during this approach in a cadaveric study [14]. Thus, the risk of blood vessels injury should be taking into account.

In case of a revision a new osteotomy might be necessary if the entire prosthesis must be substituted, with an increased risk of complications. If the damage is isolated to the polyethylene it might be possible to do the exchange through anteromedial arthrotomy or lateral approach.

A study by Usuelli et al reported the possibility of a lower infection rate in total ankle replacement trough lateral approach rather than anterior approach. The incidence was reported to be of 4.9% vs 2.9% for superficial infection and 3.7% vs 1.4% for deep infections, for the anterior and lateral approach respectively. Those differences were not found to be statistically significant [15].

The survival rate of the Zimmer TM TAR has been reported by few studies with insufficient follow-up to be representative of the effectiveness of this prosthesis.

Barg et al. reported a 93% survival at 36 months with a decrease in VAS pain score from 7.9 ± 1.3 to 0.8 ± 1.2 after surgery [16]. Maccario et al. reported a 100% survival rate at 24 month and a decrease in VAS from 7.42 to 1.42 [17]. Tan reported 20 cases of TAR through lateral approach with no failures during the first 12 months, good intra-operative alignment including 8 patients (42.1%) with a mean varus of 10.5± 4.2° and 4 (21.1%) with a mean valgus of 15.5± 8.6° preoperatively. In both groups, the angles decreased to approximately 2° postoperatively [18]. In a recent paper, Usuelli et al. reported a 98.9 % survival rate at 24 months with a rate of major complications requiring surgery of 11.2 %. We found in our patients a survival rate of 100% with a decrease in VAS from 7.81 pre-operatively to 2.29 post-operatively and major complications rate of 23.3 % [19].

In this study, four patients were younger than 40 years old. Younger patients show higher activity level which can theoretical influence polyethylene wear. Therefore, an important concern in these patients is an expected higher revision rate. In literature, there are no studies reporting high long-term survival (> 20 years) of TARs, and we therefore consider younger age a contraindication for TAR not through an absolute age limit, but by taking into consideration the need of higher survivorship expectancy of the implants. When considering a younger patient for TAR, the possibility of future revisions through a revisional arthroplasty or salvage fusion, should be discussed.

A major concern with the lateral approach TAR is associated with fibular osteotomy and ligament sectioning [20]. DeVries et al. reported in a previous study of 16 patients with Zimmer TM TAR, good overall alignment even with valgus or varus hindfoot deformity [10]. However, 4 patients (25%) experienced complications related to the fibular osteotomy (non-union, delayed union, infection). In this study, fibular nonunion was present in two patients and revisional surgery was necessary in one. We described also, the first post-operative case of ankle dislocation after TAR. Despite the challenging treatment, which included reduction and temporary fixation, good mid-term outcome was achieved at the last follow-up. Considering these results, the lateral approach provides a good visualization of the joint but the risk of fibular nonunion and instability should be kept in mind when considering this approach.

The major complication rate in this study (23.3%) was similar to previous studies with different TAR systems. Hofmann et al. reported a major complication rate of 21% with the most common cause for reoperation of gutter impingement [21]. Nunley et. al reported 17% of patients requiring additional procedures after STAR system [22]. In a previous paper about Intramedullary-Fixation Total Ankle Arthroplasty (INBONE I/II), the reoperation rate due to postoperative complications in the short-term follow-up was 24% [23].

Radiolucent lines are a cause for concern after TAR. Barg et al. reported a high rate of radiolucent lines in the tibial and talar-implant interface of 34.5% and 12.7% respectively [24]. Interestingly, we observed radiolucent lines in 10% of the patients. Longer follow-up studies are warranted to understand the real nature of this radiological signs.

The radiographic angles used to assess component’s migration were stable at the last follow-up and comparable to the results found by Haytmanek et al [25]. The authors reported a mean value of 87.5 for the α angle, 88.1 for the β angle and 17.4 for γ angle.

The clinical results of this group of patients are similar to the results for pain outcomes reported in the meta-analysis by Zaidi et al., in which the mean pooled summary VAS scores decreased from 7.4 preoperatively to 1.6 postoperatively [26]. In a previous paper by Usuelli et al. the AOFAS hindfoot score of 67 patients increased from 32.8 to 85.0 [27].

In a previous study, Hsu assessed the clinical outcomes with use of AOFAS ankle-hindfoot score after both INBONE I and II total ankle arthroplasties [23]. The mean overall improvements in the AOFAS score was 43.2 points with most of the patients very satisfied with the final result. Nunley reported a mean change in the AOFAS summary score of 52 points, after the STAR arthroplasty [22]. We report similar results with an increase from 40.95 to 86.38 with a mean increase of 45.22 (range from 26 to 57).

We acknowledge the limitations of this study due to the low number of patients, its retrospective nature and limited follow-up time.

## Conclusion

In this study, patients reported high levels of satisfaction with a low incidence of complications related to the lateral approach. Most of the patients reported an increase in the AOFAS score and a decrease in FFI-P, FFI-D and VAS for pain score.

## Abbreviations

AOFAS: American Orthopedic Foot and Ankle Society
ATFL: Anterior talofibular ligament
FFI-D: Foot Function Index- Disability
FFI-P: Foot Function Index- Pain
GCT-TS: Giant cell tumor of the tendon sheath
ROM: Range of movement
TAR: Total ankle replacement
VAS: Visual Analouge scale
Zimmer TM TAR: Zimmer Trabecular Metal Total Ankle Replacement
